# Trends over 5 Decades in U.S. Occupation-Related Physical Activity and Their Associations with Obesity

**DOI:** 10.1371/journal.pone.0019657

**Published:** 2011-05-25

**Authors:** Timothy S. Church, Diana M. Thomas, Catrine Tudor-Locke, Peter T. Katzmarzyk, Conrad P. Earnest, Ruben Q. Rodarte, Corby K. Martin, Steven N. Blair, Claude Bouchard

**Affiliations:** 1 Pennington Biomedical Research Center, Louisiana State University System, Baton Rouge, Louisiana, United States of America; 2 Department of Mathematical Sciences, Montclair State University, Montclair, New Jersey, United States of America; 3 Arnold School of Public Health, Departments of Exercise Science and Epidemiology/Biostatistics, University of South Carolina, Columbia, South Carolina United States of America; Universidad Europea de Madrid, Spain

## Abstract

**Background:**

The true causes of the obesity epidemic are not well understood and there are few longitudinal population-based data published examining this issue. The objective of this analysis was to examine trends in occupational physical activity during the past 5 decades and explore how these trends relate to concurrent changes in body weight in the U.S.

**Methodology/Principal Findings:**

Analysis of energy expenditure for occupations in U.S. private industry since 1960 using data from the U.S. Bureau of Labor Statistics. Mean body weight was derived from the U.S. National Health and Nutrition Examination Surveys (NHANES). In the early 1960's almost half the jobs in private industry in the U.S. required at least moderate intensity physical activity whereas now less than 20% demand this level of energy expenditure. Since 1960 the estimated mean daily energy expenditure due to work related physical activity has dropped by more than 100 calories in both women and men. Energy balance model predicted weights based on change in occupation-related daily energy expenditure since 1960 for each NHANES examination period closely matched the actual change in weight for 40–50 year old men and women. For example from 1960–62 to 2003–06 we estimated that the occupation-related daily energy expenditure decreased by 142 calories in men. Given a baseline weight of 76.9 kg in 1960–02, we estimated that a 142 calories reduction would result in an increase in mean weight to 89.7 kg, which closely matched the mean NHANES weight of 91.8 kg in 2003–06. The results were similar for women.

**Conclusion:**

Over the last 50 years in the U.S. we estimate that daily occupation-related energy expenditure has decreased by more than 100 calories, and this reduction in energy expenditure accounts for a significant portion of the increase in mean U.S. body weights for women and men.

## Introduction

The sharp increase in the prevalence of obesity and associated health consequences over recent decades in the U.S. are well documented [1], [2]. The causes of the ongoing obesity epidemic are not well established and despite the great economic and health care significance of the obesity epidemic there are relatively few longitudinal population-based data examining this issue. At the most basic level weight is the end-product of energy consumed and energy expended. Physical activity is the only modifiable variable contributing to total energy expended and can be segmented into occupational (i.e., work-related) and non-occupational or leisure-time physical activity.

Using a variety of data sources and statistical modeling there have been a number of papers suggesting that increased food intake is largely, if not completely, responsible for the obesity epidemic [3]–[5]. One of the arguments to support this hypothesis is that time spent in leisure-time activity has remained unchanged in recent decades leading to the conclusion that it is solely excessive caloric intake that has led to the present predicament. Even though the stability of leisure time activity argument is not compelling for a variety of reasons not to be examined here, the time spent in leisure-time physical activity represents a relatively small portion of the total hours in a week. Importantly, occupational physical activity has an even greater potential to have a significant impact on total caloric expenditure. While a common assertion is that occupational physical activity has decreased in recent decades, to our knowledge this has not been examined in detail, nor have changes in occupational physical activity been associated with changes in mean body weight or the prevalence of obesity [6].

Using nationally representative data sources we examined trends in occupational physical activity during the past 5 decades and explore how these trends relate to changes in mean body weight and the prevalence of obesity in the U.S. Having a better understanding of the relative importance of occupational physical activity in the ongoing obesity epidemic should help in the formulation of a comprehensive evidenced-based plan, with policies, strategies and tactics to combat this continuing problem.

## Methods

The prevalence of obesity (body mass index ≥30.0 kg/m^2^) in U.S. adults was derived from the 1960–62, 1971–74, 1976–80, 1988–94, 1999–2000, 2001–02, 2003–04 and 2005–06 U.S. National Health and Nutrition Examination Surveys (NHANES) [7], [8]. NHANES uses a complex, multistage, probability sampling design to select participants who are representative of the civilian, non-institutionalized U.S. population. Sample weights are assigned to each individual to represent the U.S. population and allow for the development of national prevalence figures. The prevalence of obesity for each examination period was age-adjusted by the direct method to the year 2000 U.S. Bureau of Census estimates. Mean body weight for the 1960–62, 1971–74, 1976–80, 1988–94, 1999–2002 and 2003–2006 NHANES surveys for the 40–50 year old age group were examined by gender [9], [10]. We chose this age group because it has the highest percent of employed individuals for both men and women [9]. The National Center for Health Statistics (NCHS) ethics review board approved the original survey protocols, and informed consent was obtained for all NHANES participants.

Employment data were derived from the Current Employment Statistics (CES) program for the years 1960 to 2008. The CES is a monthly survey of businesses and government agencies conducted by State employment security agencies in cooperation with the Bureau of Labor Statistics. The survey provides employment, hours and earnings estimates based on payroll records of business establishments dating back to 1939, but is limited to nonagricultural industries. Occupations are broadly categorized as goods-producing or service providing. Goods-producing further sub-categorized into mining-logging, construction and manufacturing, while service-providing occupations are further divided into the categories of trade (whole sale and retail), transportation/utilities, information, financial services, professional/business services, education/health services, leisure/hospitality and other. Agricultural employment data were derived from the Current Population Survey (CPS) which is a monthly survey of households conducted by the Bureau of Census for the Bureau of Labor Statistics. The CPS provides a comprehensive body of data on the labor force including employment, unemployment and persons not in the labor force. While CPS has agricultural employment data dating back to1940, it only has detailed occupation and industry data starting in 1983.

Median, minimum and maximum physical activity intensity (Metabolic Equivalents: METs) were assigned to each occupation based on previously published classification schemes [11]. Industries were then categorized into physical activity intensity groups based on the median METs value. The intensity categories were sedentary (<2 METs), light (2.0–2.9 METS), and moderate (3.0–5.9 METs) [12]–[14].

### Statistical Analyses

The prevalence of individuals in specific occupations by year was calculated by dividing the number of individuals in a given occupation by total U.S. occupations in private industry from CES combined with total agricultural occupations from the CPS for that year. The prevalence of individuals in sedentary, light, and moderate intensity category industries was calculated for each year. Mean occupational-related METs for each year were calculated as follows: the number of individuals employed in each occupation was multiplied by the median METs for the occupation with the product for each occupation added together and the sum divided by the total number of employed individuals for that year. To estimate daily occupation-related physical activity energy expenditure we assumed an 8 hour work day, used the mean weight from 1960–1962 NHANES survey (64.9 kg for women and 76.9 kg for men) and used the mean MET value for each year with the formula: daily caloric expenditure = hours worked×mean MET value×weight (kg) [15].

### Energy Balance Differential Equation Model

Theoretical predictions of weight gain as a result of changes in energy expenditures were computed using a validated energy balance differential equation model for weight change [16], [17]. The model is derived from the first law of thermodynamics and predicts weight change resulting from changes in energy intake and energy expenditures. The predictions account for weight dependent changes in energy expenditure through specific formulations of weight dependent terms for physical activity and resting metabolic rate.

In order to compare model predictions to the NHANES data, we determined the average weight gain in kg per kcal decrease in energy expenditure resulting from the trends in occupational activities. Specifically, after decreasing energy expenditures by increments of 50, 100, and 150 kcal/d to represent decreased work-related physical activity, the model was simulated until steady state was reached. The predicted change in weight per decrease in kcal/d energy expenditure was determined by averaging the three ratios of the change in steady state weight per 50 kcal/d. From the model men gain 0.090 kg for every kcal of energy expenditure decrease and women gain 0.092 kg for every kcal of energy expenditure decrease.

## Results

**Figure 1:**
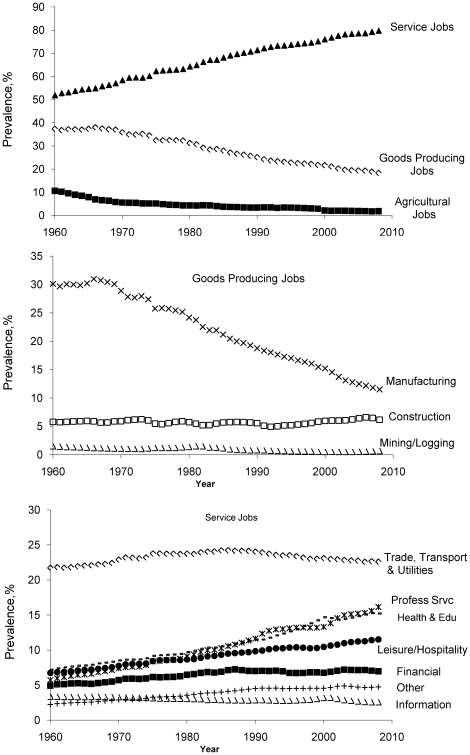
Service, goods producing and agriculture jobs in US from 1960 to 2008. The top panel depicts the prevalence of service occupations, goods producing occupations and agricultural occupations for the US from 1960 to 2008. The middle and lower panels depict the sectors within the good producing (middle panel) and service occupations (lower panel).

The top panel of Figure 1 depicts the prevalence of service occupations, goods producing occupations and agricultural occupations for the U.S. from 1960 to 2008. For both goods producing and agriculture occupations there has been a decrease over the last 5 decades while there has been a substantial increase in the prevalence of service occupations (p for trend<0.001 for each). Within the goods producing occupations (middle panel), construction has been relatively constant (p trend = 0.5) while there has been a large decrease in the prevalence of manufacturing and mining/logging occupations (p for trend<0.001 for each). Whereas more than 30% of U.S. private sector occupations were in manufacturing in the 1960's this number decreased to approximately 12% of U.S. private sector jobs by 2008. In the service occupations category (lower panel), the information category of occupation has seen a negative trend (p trend<0.001) while all other service occupations have seen an upward trend in prevalence (p≤0.01 for each). The occupation categories of professional services, health/education and leisure/hospitality in particular have seen large increases. Together, these three service occupation categories made up approximately 20% of U.S. occupations in the early 1960's and by the year 2008 they represented 43% of U.S. occupations.

Table 1 summarizes the estimated median and range of physical activity intensity (METs) as well as the estimated caloric expenditure of each occupation. Mining/logging, construction and manufacturing all qualified as moderate intensity activity. All service occupation sectors were either sedentary or light intensity activity.

**Table 1:** Estimated median and range of physical activity intensity (METs) as well as the estimated caloric expenditure of each occupation.

	METsMedian (min, max)	ActivityCategory
**Farm Jobs**	3.0 (2.5, 4.5)	Moderate
**Goods-Producing**		
Mining and logging	3.8 (3.0, 8.0)	Moderate
Construction	4.0 (1.5, 7.5)	Moderate
Manufacturing	3.0 (1.5, 4.0)	Moderate
**Service-Providing**		
Trade (wholesale & retail), transportation, and utilities	2.0 (1.5, 3.0)	Light
Information	1.5 (1.5, 1.5)	Sedentary
Financial activities	1.5 (1.5, 1.5)	Sedentary
Professional and business services	1.5 (1.5, 2.0)	Sedentary
Education and health services	2.5 (1.5, 4.0)	Light
Leisure and hospitality	2.5 (1.5, 3.5)	Light
Other services	2.5 (1.5, 3.0)	Light

**Figure 2:**
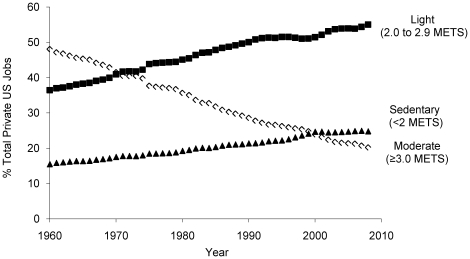
Trends in the prevalence of sedentary, light and moderate intensity occupations from 1960 to 2008.

**Figure 3:**
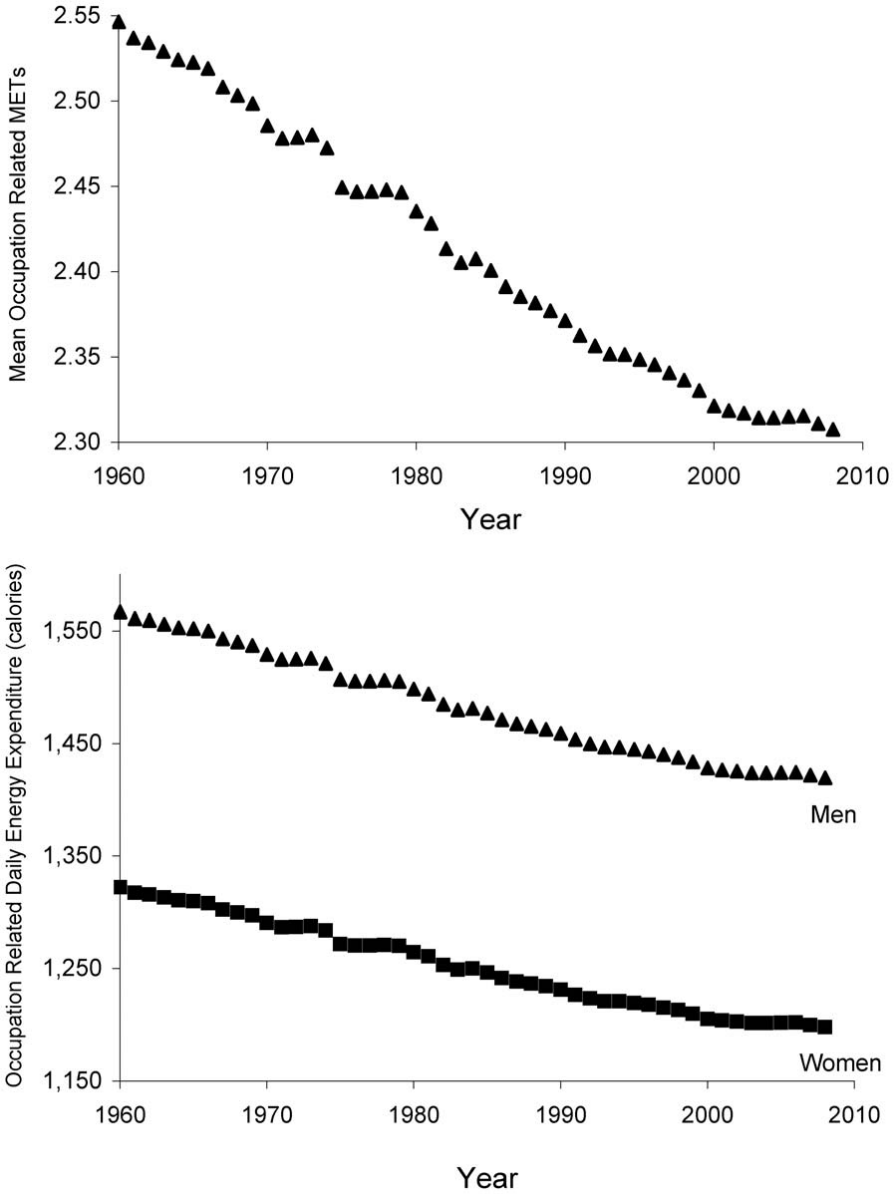
Occupational METs and energy expenditure since 1960. The upper panel of Figure 3 plots the mean occupation-related METs since 1960 and the lower panel presents the mean occupational daily energy expenditure in men and women since 1960.

Figure 2 presents the trends in the prevalence of sedentary, light, and moderate intensity occupations from 1960 to 2008. While there has been a steady increase in the prevalence of sedentary and light intensity physical activity occupations since 1960, the prevalence of moderate intensity physical activity occupations has decreased from 48% in 1960 to 20% in 2008 (p trend<0.001 for each). Figure 3 plots the mean occupation-related METs (top panel) and the associated change in occupation-related daily caloric expenditure for women and men (bottom panel). There was a steep decline in mean occupation-related METs and consequently mean occupation-related physical activity energy expenditure from 1960 to 2008 (p trend<0.001 for each). From 1960 to 2008 there was an approximate drop in occupation-related daily energy expenditure of 140 calories for men and 124 calories for women.

**Figure 4:**
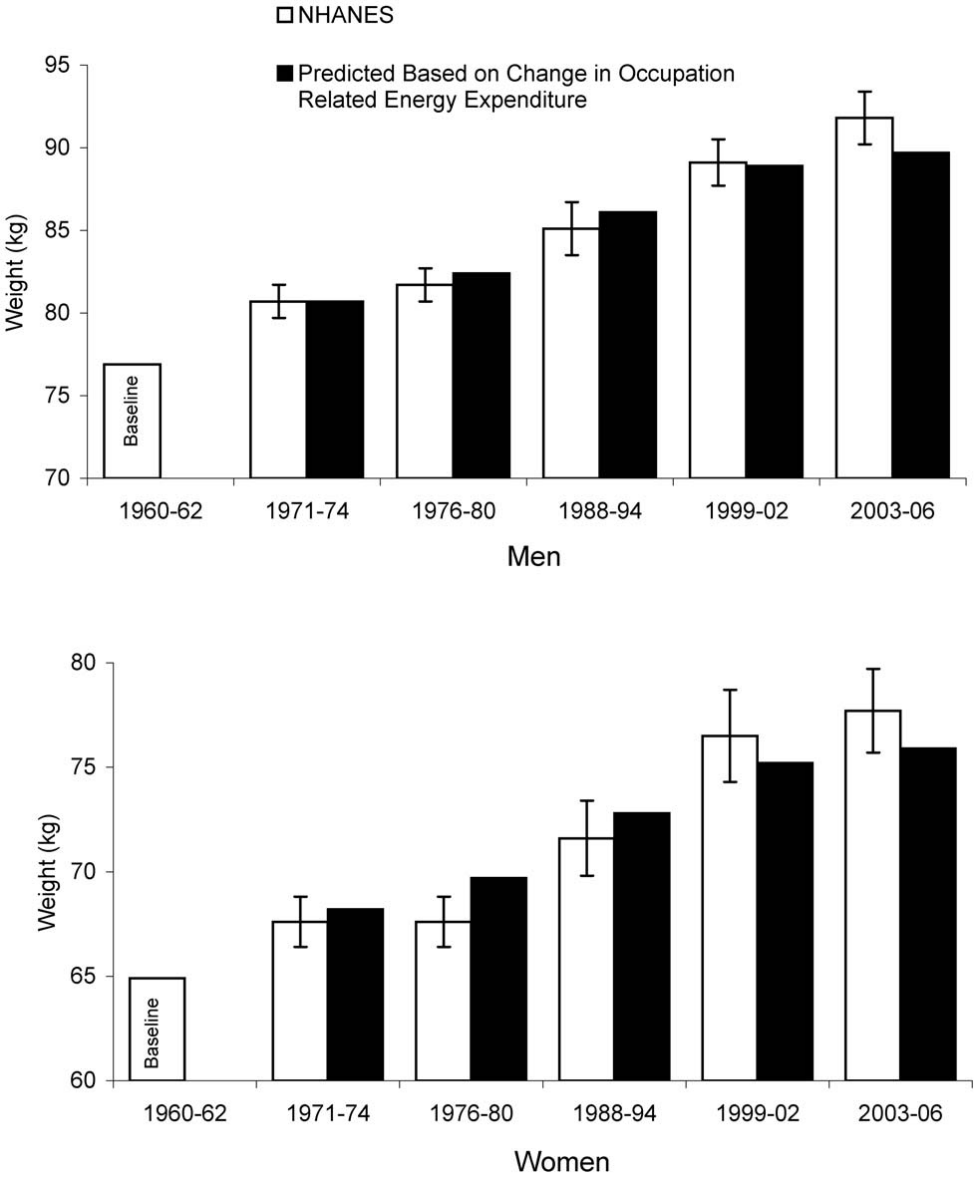
Predicted weights and NHANES weights. Figure 4 presents the energy balance model predicted mean U.S. body weight based on change in occupation related daily energy expenditure since 1960 compared to mean U.S. weight gain based on the NHANES examination periods for 40–50 year old men (left panel) and women (right panel).

Figure 4 presents the energy balance model predicted weights based on change in occupation-related daily energy expenditure since 1960 for each NHANES examination period compared to the actual change in weight for 40–50 year old men (top panel) and women (lower panel). The energy balance model predictions assumed a 5 day work week. For both men and women the predicted weight based on changes in occupational energy expenditure closely matches the NHANES weight for each examination period.

## Discussion

Over the last 50 years in the U.S. there has been a progressive decrease in the percent of individuals employed in goods producing and agriculture occupations whereas there has been an increase in the percent of individuals employed in service occupations. This has resulted in a shift away from occupations that require moderate intensity physical activity to occupations that are largely composed of sitting and sedentary behavior. In the early 1960's almost half of private industry occupations in the U.S. required at least moderate intensity physical activity and now less than 20% demand this level of activity. We estimate that daily occupation-related energy expenditure has decreased by more than 100 calories in both women and men, and further, this reduction in occupational energy expenditure accounts for a large portion of the observed increase in mean U.S. weight over the last 5 decades.

Examining secular changes in total daily physical activity is a complex endeavor. Two examples include the observation that time spent in recreational activities has increased but so has time spent watching TV. At the same time, time spent on household work has greatly decreased in women but slightly increased in men [6]. Dissecting the relative importance of each of these activities of daily living on body weight is challenging. Here we chose to focus on occupation-related energy expenditure because time spent at work represents the largest segment of waking hours for most people in the age range we studied. It is important to note that we examined the prevalence of different occupations, not absolute number of jobs in a given occupation. This is important because the workforce is not a static population and over the last 50 years the prevalence of Americans in the Labor Force has increased from approximately 40% to 50% [18]. One of the driving forces behind the increased prevalence of working Americans is the increase of women in the work force. In 1970 43% percent of women were in the labor force and by 2007 this increased to 60% of women. This fact may also explain why occupation-related energy expenditure estimated a higher mean weight during the years of 1971 to 1994 but closely matched the mean weight of women from NHANES during the period of 1999–2002 [18].

Given that it is unlikely that there will be a return to occupations that demand moderate levels of physical activity; our findings provide further strong evidence of the public health importance of promoting physically active lifestyles outside of the work day. Our estimation of a reduction of more than 100 calories per day in occupation-related energy expenditure over the last 50 years would have been adequately compensated for by meeting the 2008 federal physical activity recommendations of 150 minutes per week of moderate intensity activity or 75 minutes per week of vigorous intensity activity [13]. While it is often noted that the prevalence of Americans who achieve this recommendation has been constant over recent decades, the fact remains that based on self-report data only 1 in 4 Americans achieve this level [19]. It is important to note that when physical activity is assessed with accelerometers the number of Americans that achieve the physical activity recommendations falls to 1 in 20 [20]. Thus since energy expenditure has largely been removed from the work place the relative importance of leisure-time physical activity has increased and should be a major focus of public health interventions and research.

Based on estimated caloric consumption from food production and food disappearance (food waste) estimates, previous reports have concluded that increased caloric consumption could account for most, if not all, of the weight gained at a population level in the U.S. [3]–[5]. Nonetheless, a recently validated differential equation model was used to identify a conservative lower bound for the amount of food waste in the U.S. [21]. This analysis determined that prior estimates of national food waste were grossly underestimated; indicating that the national average caloric intake was lower than previously estimated. These results and the results of the present study indicate that changes in caloric intake cannot solely account for the observed trends in national weight gain.

Our analysis has strengths and weaknesses that deserve mention. A major strength of this analysis is that for both the U.S. obesity and occupation data we used nationally representative databases. Further, we used a previously published and well-recognized classification system to assign physical activity intensity levels to each occupation category thus minimizing the possibility of misclassifying occupation-related physical activity intensity [11]. However, we used the same physical activity classifications across the 5 decades examined and in doing so we did not take into account changes in technology that have reduced physical labor. While technological advances have greatly reduced the physical labor associated with most manufacturing operations this phenomenon would drive our results towards the null and thus we may be underestimating the true loss of moderate intensity occupations in the work force. Our analysis was focused on type of occupation, and there are many aspects of occupation related daily energy expenditure we did not examine such as mode of travel to work, total sitting time and stair usage. Another weakness of our analysis is that not all agricultural or goods producing occupations are associated with higher levels of physical activity and conversely some service-related occupations are associated with higher levels of physical activity. However, there are no adequate data to examine this level of detail and we were very conservative in assigning MET values in order to minimize the effect of such misclassification within occupation types.

### Conclusions

Over the last 50 years in the U.S. there has been a progressive decrease in the percent of individuals employed in occupations that require moderate intensity physical activity. We estimate that daily occupation-related energy expenditure has decreased by more than 100 calories, and this reduction in energy expenditure accounts for a significant portion of the increase in mean U.S. body weight for women and men over the last 5 decades.
